# Long-term social and professional outcomes in adults after pediatric kidney failure

**DOI:** 10.1007/s00467-023-06029-2

**Published:** 2023-06-17

**Authors:** Guido F. Laube, Marc-Andrea Heinzelmann, Katharina Roser, Claudia E. Kuehni, Luzius Mader, on behalf of the Swiss Pediatric Renal Registry (SPRR) group

**Affiliations:** 1 Department of Pediatrics, Hospital Baden, Baden, Switzerland; 2 Swiss Pediatric Renal Registry, Child and Adolescent Health Research Group, Institute of Social and Preventive Medicine, University of Bern, Bern, Switzerland; 3 Child and Adolescent Health Research Group, Institute of Social and Preventive Medicine, University of Bern, Bern, Switzerland; 4 Faculty of Health Sciences and Medicine, University of Lucerne, Lucerne, Switzerland; 5 Department of Pediatrics, University Children’s Hospital Bern, Bern, Switzerland; 6 Cancer Registry Bern-Solothurn, University of Bern, Bern, Switzerland

**Keywords:** Kidney replacement therapy, Pediatric kidney failure, Partner relationship, Living situation, Education, Employment

## Abstract

**Background:**

Little is known about the long-term social and professional outcomes in adults after pediatric kidney replacement therapy (KRT). In this study, we described social and professional outcomes of adults after kidney failure during childhood and compared these outcomes with the general population.

**Methods:**

We sent a questionnaire to 143 individuals registered in the Swiss Pediatric Renal Registry (SPRR) with KRT starting before the age of 18 years. In the questionnaire, we assessed social (partner relationship, living situation, having children) and professional (education, employment) outcomes. Logistic regression models adjusted for age at study and sex were used to compare outcomes with a representative sample of the Swiss general population and to identify socio-demographic and clinical characteristics associated with adverse outcomes.

**Results:**

Our study included 80 patients (response rate 56%) with a mean age of 39 years (range 19–63). Compared to the general population, study participants were more likely to not have a partner (OR = 3.7, 95%CI 2.3–5.9), live alone (OR = 2.5, 95%CI 1.5–4.1), not have children (OR = 6.8, 95%CI 3.3–14.0), and be unemployed (OR = 3.9, 95%CI 1.8–8.6). No differences were found for educational achievement (*p* = 0.876). Participants on dialysis at time of study were more often unemployed compared to transplanted participants (OR = 5.0, 95%CI 1.2–21.4) and participants with > 1 kidney transplantation more often had a lower education (OR = 3.2, 95%CI 1.0–10.2).

**Conclusions:**

Adults after pediatric kidney failure are at risk to experience adverse social and professional outcomes. Increased awareness among healthcare professionals and additional psycho-social support could contribute to mitigate those risks.

**Graphical abstract:**

**Figure:**
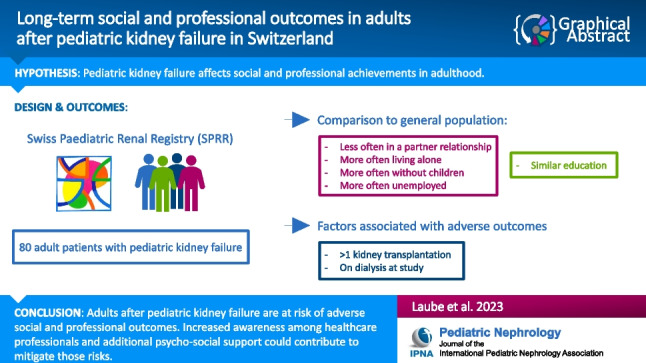
A higher resolution version of the Graphical abstract is available as Supplementary information

**Supplementary Information:**

The online version contains supplementary material available at 10.1007/s00467-023-06029-2.

## Introduction

Kidney replacement therapy (KRT) consisting of peritoneal dialysis, hemodialysis, and kidney transplantation is the treatment of choice in children and adolescents with kidney failure. Based on steadily improving surgical techniques and immunosuppressive treatments, patient and graft survival have significantly improved during the last decades. Clinical outcomes after pediatric kidney transplantation are therefore favorable and patients reach adulthood [1, 2]. However, cardiovascular disease, malignancies, growth retardation, and side effects of immunosuppressive treatment should be taken into consideration while caring for these patients [3]. After pediatric kidney transplantation, health-related quality of life (HRQoL) rated by children and adolescents themselves is similar to healthy peers, although parents indicated impaired HRQoL of their children [4]. We recently showed that adults after pediatric kidney transplantation have lower physical HRQoL than the general population while mental HRQoL was similar [5].

The burden of being chronically ill since childhood or adolescence raises the question of social and professional outcomes in adulthood. Previous publications indicate lower social and professional achievements in adults 20 to 30 years after KRT programs had been initiated [6-16]. Studies vary on number of participants, age at time of survey and country of origin. Major impairments were found for partnership, living independency, educational level, and occupational activity. It seems that, despite excellent medical improvements during the last decades, the final goal of satisfying social and professional outcomes has not been achieved yet [6-11].

Based on the Swiss Pediatric Renal Registry (SPRR) [17], which includes data about children with kidney failure in Switzerland since 1970, we evaluated long-term social and professional outcomes. Specifically, we compared social (partner relationship, living situation, having children) and professional (educational achievement, employment status) outcomes of adults, who started continuous KRT before the age of 18 years in Switzerland, with the general population, and investigated socio-demographic and clinical characteristics associated with adverse social and professional outcomes.

## Methods

### Design, study population, and research setting

In this cross-sectional study, we included patients registered in the SPRR fulfilling the following inclusion criteria: (1) continuous KRT before the age of 18 years (kidney transplantation before the age of 18 years or dialysis before the age of 18 years followed by a kidney transplantation after the age of 18 years), (2) aged ≥ 18 years at study, (3) alive, and (4) formerly treated in a German-speaking pediatric hospital in Switzerland. We focused on the language region covering the largest number of patients due to feasibility reasons [5] and limited resources for translating and back-translating the study material in this unfunded study. We sent a paper-based questionnaire assessing a broad range of long-term outcomes to all eligible individuals between 2021 and 2022 with the option to complete it online. The questionnaire was based on a previous pilot study and the baseline questionnaire of the Swiss Childhood Cancer Survivor Study [18]. It covered the kidney disease and treatments, somatic health, current medication, quality of life, social support, psychological well-being, health behaviors, and information about the social and professional situation [5]. Non-responders received up to two reminders approximately 4 and 12 weeks after initial contact. Ethical approval of the SPRR was granted by the Ethics Committee of the Canton of Bern (140/2015). Informed consent was provided by all study participants.

As a comparison group, we used participants from a population-based survey consisting of a random representative sample of the Swiss general population (regarding age, sex, and language region) from the Swiss Federal Statistical Office which included individuals between 18 and 75 years of age in 2015. We weighted the study participants (responders to the outcome questions) regarding age, sex, and nationality (Swiss, other) using the distributions of these characteristics in the entire eligible sample obtained from the Swiss Federal Statistical Office [19]. Eligible persons were contacted between 2015 and 2016 with study information 2 weeks prior to sending a paper-based questionnaire, and one reminder to non-responders after 4 weeks. Ethical approval for the general population sample was granted through the Ethics Committee of Northwest and Central Switzerland (EKNZ 2015–075).

### Social and professional outcome measures

For the SPRR study population and the general population, we assessed the following social and professional outcomes in the questionnaire: partner relationship (no; yes), living situation (living alone; not living alone), having children (no; yes), educational achievement (dichotomized into compulsory schooling or vocational training; upper secondary or university education) [20], and employment status (unemployed; employed or studying). SPRR participants were also asked whether they attended a special school (no; yes), whether they received educational support during hospital stays by means of tutoring or computer-based learning (no; yes), and whether this hospital-based educational support was sufficient to allow them to follow regular school activities (no; yes).

### Socio-demographic and clinical characteristics

For SPRR participants and the general population, we assessed age at study (continuous) and sex (male; female) in the questionnaire. For the SPRR population, we additionally obtained the following clinical characteristics from the SPRR: type of kidney disease, age at first KRT (< 10 years; ≥ 10 years), and duration of KRT (< 25 years; ≥ 25 years). Type of kidney disease was categorized into congenital anomalies of the kidney and urinary tract, monogenetic hereditary diseases, and acquired diseases [17]. We assessed type of KRT at study (dialysis, transplantation), number of transplants (1 transplant, > 1 transplant), and height (in cm) in the questionnaire.

### Statistical analysis

First, we tabulated characteristics of the SPRR study population comparing participants and non-participants. Second, we described social and professional outcomes of SPRR participants and used logistic regression models adjusted for age at study and sex to estimate odds ratios (OR) and corresponding two-sided 95% confidence intervals (CI) comparing SPRR participants to the general population. Third, we used logistic regression models (crude and adjusted for age at study and sex) to identify associations between social and professional outcomes (partner relationship, living situation, having children, educational achievement, employment status), and socio-demographic characteristics, clinical characteristics, attendance of special school, and educational support during hospital stays. All statistical analyses were based on complete cases and performed using Stata version 15.1 (StataCorp. 2017. *Stata Statistical Software: Release 15*. College Station, TX: StataCorp LLC).

## Results

### Study participants and their characteristics

**Fig. 1:**
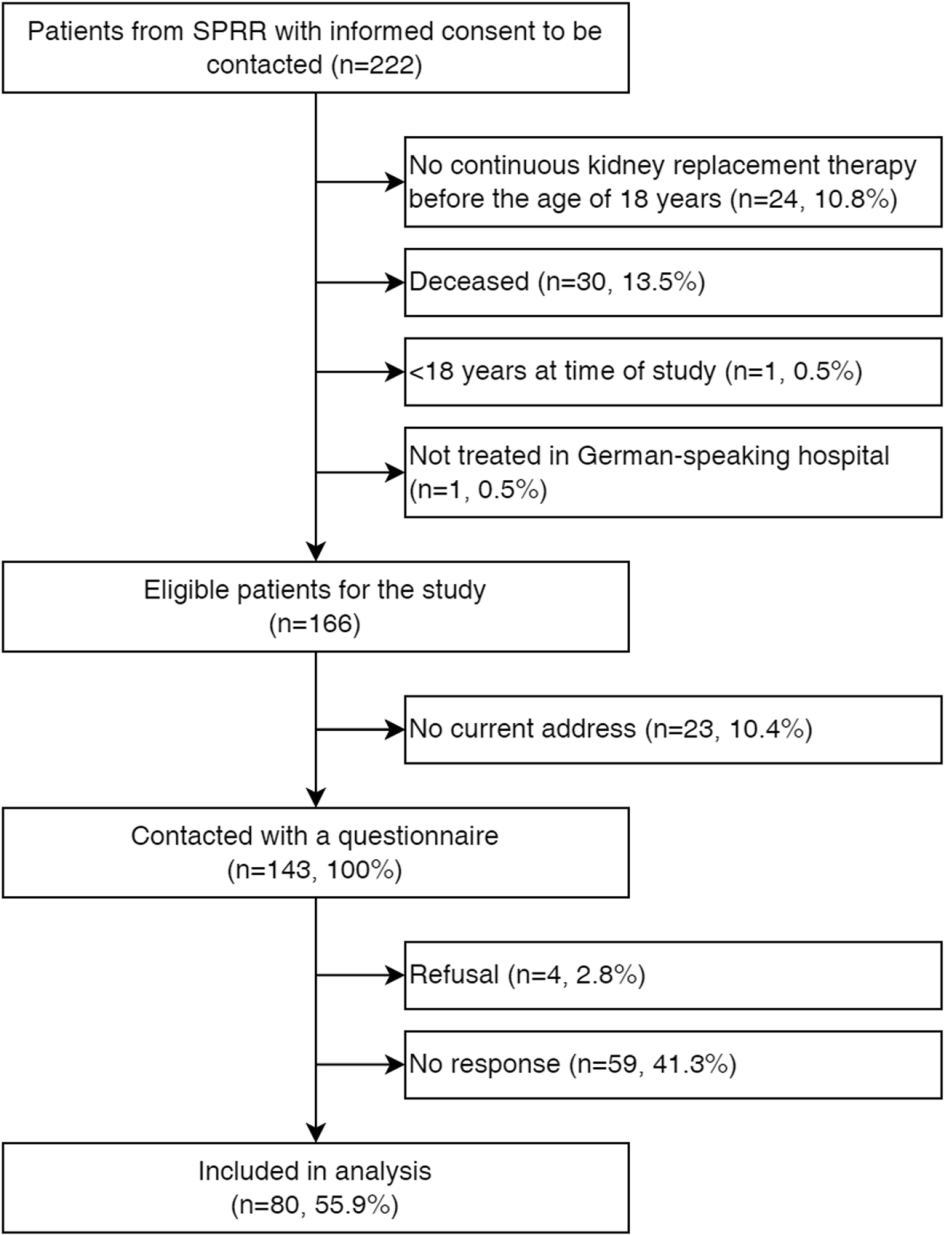
Flowchart of the study population

Of 166 eligible patients identified in the SPRR, 143 received the questionnaire (Fig. 1). Of those, 4 patients (3%) declined to participate, 59 (41%) did not respond, and 80 (56%) completed the questionnaire. One third (34%) completed the questionnaire online and 66% used the paper-based version. Socio-demographic and clinical characteristics are presented in Table 1. The mean age at study was 39 years (range: 19–63) and 56% of participants were male. The majority of participants were diagnosed with a monogenetic hereditary disease (44%). The mean age at first KRT was 10 years (range 0–18) with an average duration of KRT of 28 years (range 11–49). All participants had been transplanted at least once. At time of study, most participants had a functioning transplant (84%). The mean height was 165 cm (range 125–182). Using information from the SPRR, we found no differences between participants and non-participants for age at study, sex, type of kidney disease, age at first KRT, and duration of KRT (all *p* > 0.05; Online Resource 1).

**Table 1:** Characteristics of SPRR patients who completed the questionnaire (*n* = 80)

	n	%
*Mean age at study in years (range)*	39 (19–63)	
*Sex*		
Male	45	56
Female	35	44
*Type of kidney disease*		
Congenital anomalies of kidney and urinary tract	29	36
Monogenetic hereditary diseases	35	44
Acquired diseases	16	20
*Age at first KRT*		
Mean in years (range)	10 (0–18)	
< 10 years	32	40
≥ 10 years	48	60
*Duration of KRT*		
Mean in years (range)	28 (11–49)	
< 25 years	32	40
≥ 25 years	48	60
*Type of KRT at study*		
Transplantation	67	84
Dialysis	10	13
Missing	3	4
*Number of transplants*		
1 transplant	45	56
2 transplants	26	33
3 transplants	9	11
*Mean height in cm (range; SD)*	165 (125–182; 10)	
Males	169 (125–182; 10)	
Females	160 (145–179; 8)	

*Abbreviations: KRT*, kidney replacement therapy

The sample of the general population consisted of 1255 individuals representative for the Swiss general population in terms of age, sex, and nationality [19]. The mean age of the general population sample was 49 years (range 18–76) and 42% were male.

### Social and professional outcomes and comparison with the general population

Social and professional outcomes of SPRR participants in comparison with the general population are displayed in Table 2. A partner relationship was reported by 45% SPRR participants and 33% were living alone. Adjusted for age and sex, SPRR participants were nearly 4 times more likely not to have a partner (OR = 3.7, 95%CI 2.3–5.9) and 2.5 times more likely to live alone compared to the general population (OR = 2.5, 95%CI 1.5–4.1). Few SPRR participants reported having children and compared to the general population were 7 times more likely not to have children (OR = 6.8, 95%CI 3.3–14.0). SPRR participants had similar educational achievements compared to the general population. Twenty-six percent of SPRR participants had attended a special school. About half (51%) reported that they received educational support during hospital stays of which the majority reported that this support was sufficient to follow regular school activities. Twenty-five percent of SPRR participants were unemployed, resulting in being 4 times more likely to be unemployed compared to the general population (OR = 3.9, 95%CI 1.8–8.6).

**Table 2:** Social and professional outcomes of SPRR participants in comparison with the general population

	SPRR participants (*n* = 80)		General population (*n* = 1255)^a^		
	*n*	%	%	OR (95%CI)^c,d^	*p*-value
*Partner relationship*				**3.7 (2.3–5.9)**	** < 0.001**
No	43	54	22		
Yes	36	45	78		
Missing	1	1	-		
*Living situation*				**2.5 (1.5–4.1)**	**0.001**
Living alone	26	33	18		
Not living alone	54	67	82		
*Having children*				**6.8 (3.3–14.0)**	** < 0.001**
No	69	86	41		
Yes	11	14	59		
*Educational achievement*				1.0 (0.6–1.7)	0.876
Compulsory schooling/ vocational training	41	51	55		
Upper secondary/ university education	35	44	45		
Missing	4	5	-		
*Attendance of special school*				-	-
No	58	73	-		
Yes	21	26	-		
Missing	1	1	-		
*Educational support during hospital stays*				-	-
No	39	49	-		
Yes	41	51	-		
*Educational support during hospital stays sufficient to follow regular school*^*e*^				-	-
No	11	27	-		
Yes	27	66	-		
Missing	3	7	-		
*Employment status*				**3.9 (1.8–8.6)**	**0.001**
Employed or studying	60	75	77		
Unemployed	20	25	23		

*Abbreviations: CI*, confidence interval; *OR*, odds ratio; *SPRR*, Swiss Paediatric Renal Registry

Statistically significant associations at *p* < 0.05 are highlighted in bold

^a^Weighted to be representative for the Swiss general population in terms of age, sex, and nationality (Swiss, other)

^c^Odds ratio from logistic regression models comparing SPRR participants to representative sample of the Swiss general population (reference group): OR > 1 indicates a higher likelihood of not having a partner relationship, living alone, not having children, having a lower educational achievement, or being unemployed in SPRR participants. OR < 1 indicates a lower likelihood of not having a partner relationship, living alone, not having children, having a lower education, or being unemployed in SPRR participants

^d^Adjusted for age at study and sex

^e^Only assessed among participants that received educational support during hospital stays

### Determinants of adverse social and professional outcomes

Adjusted for age at study and sex, we found that SPRR participants who had attended a special school were less likely to report a partner relationship (OR = 3.7, 95%CI 1.2–11.7; Table 3) and were more likely to have a lower educational achievement (OR = 22.4, 95%CI 2.8–183.3). No significant associations were identified between educational support during hospital stays and social and professional outcomes. In terms of clinical characteristics, SPRR participants receiving dialysis at time of the study were more likely to be unemployed compared to transplanted participants (OR = 5.0, 95%CI 1.2–21.4). SPRR participants with > 1 kidney transplantation were more likely to report a lower educational achievement (OR = 3.2, 95%CI 1.0–10.2). We found no associations between adverse social and professional outcomes and type of kidney disease, age at first KRT, duration of KRT, number of transplants, or participants’ height (Table 3, Online Resource 2).

**Table 3:** Socio-demographic and clinical determinants of adverse social and professional outcomes from logistic regression models adjusted for age at study and sex in SPRR participants

SPRR study population (*n* = 80)					
Explanatory variables	Partner relationship (*n* = 79)	Living situation (*n* = 80)	Having children (*n* = 80)	Educational achievement (*n* = 76)	Employment status (*n* = 80)
	OR (95%CI)^a,b^	OR (95%CI)^a,b^	OR (95%CI)^a,b^	OR (95%CI)^a,b^	OR (95%CI)^a,b^
*Age at study (years)*	0.98 (0.94–1.03)	1.02 (0.98–1.07)	0.95 (0.89–1.01)	0.98 (0.94–1.02)	1.03 (0.98–1.08)
*Sex*					
Male	ref	ref	ref	ref	ref
Female	0.8 (0.3–2.1)	0.5 (0.2–1.4)	0.4 (0.1–1.7)	0.9 (0.4–2.2)	1.0 (0.3–2.8)
*Attendance of special school*					
No	ref	ref	ref	ref	ref
Yes	**3.7 (1.2–11.7)**	0.6 (0.2–2.0)	1.3 (0.2–7.1)	**22.4 (2.8–183.3)**	2.2 (0.7–7.0)
*Educational support during hospital stay*					
No	ref	ref	ref	ref	ref
Yes	0.6 (0.2–1.6)	0.8 (0.3–2.3)	0.5 (0.1–2.4)	2.0 (0.7–5.5)	0.4 (0.1–1.2)
*Type of kidney disease*					
CAKUT	ref	ref	ref	ref	ref
Monogenetic hereditary diseases	1.0 (0.4–2.8)	0.5 (0.2–1.3)	1.2 (0.3–5.0)	0.6 (0.2–1.7)	0.5 (0.2–1.8)
Acquired diseases	0.9 (0.2–3.3)	0.5 (0.1–2.1)	n.e	0.6 (0.2–2.5)	2.1 (0.5–8.7)
*Age at first KRT*					
< 10 years	ref	ref	ref	ref	ref
≥ 10 years	0.9 (0.3–2.8)	0.5 (0.1–1.5)	0.5 (0.1–2.9)	0.6 (0.2–1.7)	1.0 (0.3–3.4)
*Duration of KRT*					
< 25 years	ref	ref	ref	ref	ref
≥ 25 years	0.8 (0.3–2.5)	1.5 (0.4–5.2)	7.4 (0.8–69.4)	1.7 (0.5–5.5)	0.5 (0.1–2.2)
*Type of KRT at study*					
Transplantation	ref	ref	ref	ref	ref
Dialysis	0.8 (0.2–3.0)	0.7 (0.1–3.0)	2.4 (0.2–25.8)	2.6 (0.6–11.6)	**5.0 (1.2–21.4)**
*Number of transplants*					
1 transplant	ref	ref	ref	ref	ref
> 1 transplant	0.7 (0.3–2.0)	0.9 (0.3–2.6)	0.3 (0.1–1.4)	**3.2 (1.0–10.2)**	1.1 (0.3–3.5)
* Height [in cm]*	0.97 (0.92–1.02)	1.03 (0.97–1.09)	1.0 (0.94–1.08)	0.96 (0.91–1.02)	0.94 (0.89–1.00)

*Abbreviations: CAKUT*, congenital anomalies of the kidney/urinary tract; *CI*, confidence interval; *KRT*, kidney replacement therapy; *n.e*., not estimated due to empty cells; *OR*, odds ratio; *ref*, reference group; *SPRR*, Swiss Paediatric Renal Registry

Statistically significant associations at *p* < 0.05 are highlighted in bold

^a^Odds ratio from logistic regression models: OR > 1 indicates a higher likelihood of not having a partner relationship, living alone, not having children, having a lower education, or being unemployed. OR < 1 indicates a lower likelihood of not having a partner relationship, living alone, not having children, having a lower education, or being unemployed

^b^Adjusted for age at study and sex

## Discussion

This study showed that adults who experienced pediatric kidney failure are at risk of adverse social and professional outcomes in the long-term. Compared to the general population, they were less likely to have a partner relationship or to have children and more likely to live alone and to be unemployed. In contrast, educational achievement was comparable to the general population. Being on dialysis and having received more than one kidney transplantation were the most important clinical determinants of adverse social and professional outcomes.

Comparison of family life needs to be done carefully, especially in terms of definition of partnership. Asking adult patients about marriage excludes those who live together with a partner without having a marital status. The percentages of pediatric transplanted adult patients being married were 17%, 23%, and 27% in studies from Japan, Portugal, and France, respectively [7, 11, 13], whereas 34% were living with a partner in a Dutch study [9] and 31% in a French study [6]. Compared to that, the 45% of SPRR participants who reported having a partner is high, but we also had the highest mean age at study (39 years, compared to 27–32 years in other studies). Mellerio and colleagues suggested that living in a partnership might simply be delayed in comparison with same aged peers [6]. Adjusting for age and sex we found that participants were nearly 4 times more likely not to have a partner than the general population, which is remarkable. Additionally, we found that patients who had attended a special school were less likely to report a partner relationship. However, special schooling is often required based on severity of disease and syndromic diseases. This should be taken into consideration regarding causality. However, education and schooling seem to be an important basis for a social life, a satisfactory family situation and good HRQoL [5].

One-third of our cohort was living alone, which is 2.5 times higher compared to the Swiss general population. This result is similar to other observations and indicates that adults after pediatric kidney failure may experience difficulties pursuing a satisfactory social life [6, 9, 12, 13]. Living alone is further associated with poorer health [21]. Percentage of patients living alone was even higher with up to 50% in other studies [22, 23], in particular when kidney transplantation had been performed during the beginnings of the pediatric kidney transplantation era (1973–1985) [7]. Only 14% of our participants had children, which reflects a 7 times higher likelihood of not having children compared to the Swiss general population. This is in line with most previous studies [6, 7, 9, 13]. One reason for this might be the low number of patients living in a partner relationship. However, we need to recall that current studies included patients initially treated 20–40 years earlier, when surgical techniques and immunosuppressive treatment differed from today’s options. Patients were confronted with longer dialysis duration, longer waiting time for a transplant, and less developed immunosuppressive treatment. Currently treated children and adolescents hopefully will experience fewer social difficulties in the future.

SPRR participants showed similar educational achievements compared to the Swiss general population. Twenty-six percent attended a special school and 51% received educational support during hospital stays, of whom 66% indicated this support being sufficient to follow regular school. Attendance of a special school was strongly associated with a low final educational achievement. In terms of causality, this association may rather be explained by the severity of the underlying disease (e.g., syndromes affecting cognition) that influence the need of special schooling and in turn, later educational achievement. No association was identified between educational support during hospital stays and social and professional outcomes; however, statistical power was limited by the small sample size. Only few studies have analyzed educational achievement in adults after pediatric kidney failure in comparison with the general population. A Portuguese study indicated academic qualifications not being different when compared with the national average [13], whereas a French study by Broyer and colleagues showed that the educational level was lower when compared with the French average [7]. In some cases, dialysis, hospitalizations, and medical complications affected regular school attendance, which can explain low educational achievement. We found that SPRR participants with more than one kidney transplantation were more likely to report a lower educational achievement, reflecting the length and intensity of the kidney disease as a factor influencing long-term educational achievement.

Twenty-five percent of our cohort were unemployed as adults, resulting in a 4 times higher likelihood to be unemployed in comparison to the general population. SPRR patients receiving dialysis at time of the study were more likely to be unemployed compared to transplanted participants, indicating the burden of dialysis in terms of professional activity. The level of unemployment in our study was high, but fits with other European study results [6, 9, 13]. These results overall underline the difficulties patients with a chronic disease face in terms of integration in the professional market. Employers might fear frequent clinic visits, hospitalizations, and dialysis sessions of their potential employees and therefore are hesitant to employ adult persons with KRT. Especially patients on dialysis are at risk of being unemployed. In a Dutch cohort, van Manen and colleagues revealed a loss of permanent jobs of approximately 20% within 1 year after dialysis started [24]. From a historical point of view, we can state that during the last decades unemployment in these patients had decreased. In 1996, Gamperli and colleagues showed a decrease of unemployment from 61 to 38% over 10 years in Switzerland. Therefore, this further reduction to 25% shown in our study is an encouraging improvement, although there is still a large gap to fill [25]. There is an urgent need of consequently integrating patients with chronic diseases within the social and professional life, especially within the labor market.

The main limitation of our study is the relatively small sample size limiting statistical power for subgroup comparisons and analysis of risk factors, particularly regarding clinical characteristics. Although we found no differences when comparing participants and non-participants, our findings might be affected by selection or non-response bias. Additionally, our study was restricted to patients treated in a clinic in the German-speaking part of Switzerland reflecting most patients with pediatric kidney failure in Switzerland; however, they may not be totally representative for all patients. Another limitation may be reporting bias. Participants with better social and professional outcomes may have been more likely to complete the questionnaire or participants may have reported more favorable outcomes due to social desirability [26]. Our study therefore may have underestimated the impact of pediatric kidney failure on social and professional outcomes in adulthood. Furthermore, we collected data for SPRR patients between 2021 and 2022, whereas comparison data were obtained in 2015. Therefore, differences caused by broader circumstances (e.g., COVID-19 pandemic) could not be avoided or accounted for. Finally, no in-depth information on our outcomes was available (e.g., relationship satisfaction, individual preferences for having children, reasons for unemployment) emphasizing the need for future research. A major strength of our study is the use of a representative sample of the Swiss general population who completed the same questions on social and professional outcomes as a comparison group. Our study further included adults after pediatric kidney failure on average 39 years of age providing long-term follow-up information on this population. Another strength refers to the use of high-quality clinical information based on medical records from the SPRR.

In conclusion, adults after pediatric kidney failure are at risk to experience adverse social and professional outcomes. Although educational levels were within normal range, the number of unemployed participants was high. Additionally, we state a significant implication in social, especially familial, life in these patients. Increased awareness among healthcare professionals and additional psycho-social support could contribute to mitigate those risks. More studies evaluating social and professional outcomes are required to better understand the lifelong sequelae of adults after pediatric kidney failure. This will help healthcare professionals optimizing patient care, especially in terms of social support.

### Supplementary Information

Below is the link to the electronic supplementary material.

Graphical abstract (PPTX 339 KB)

Supplementary file 1 (DOCX 66 KB)

## Data Availability

The data that support the information of this manuscript were accessed on secured servers of the Institute of Social and Preventive Medicine at the University of Bern. Data can only be made available for researchers who fulfil the respective legal requirements. All data requests should be communicated to the corresponding author.
