# Supplementary material for: Long-term social and professional outcomes in adults after pediatric kidney failure
Source: Pediatr Nephrol. 2023 Jun 17;38(11):3769–77. doi: 10.1007/s00467-023-06029-2 (PMC10514133; doi:10.1007/s00467-023-06029-2)
Supplement: Supplementary file 1 — Graphical abstract (PPTX 339 KB) [file 467_2023_6029_MOESM1_ESM.pptx]

## Slide 1
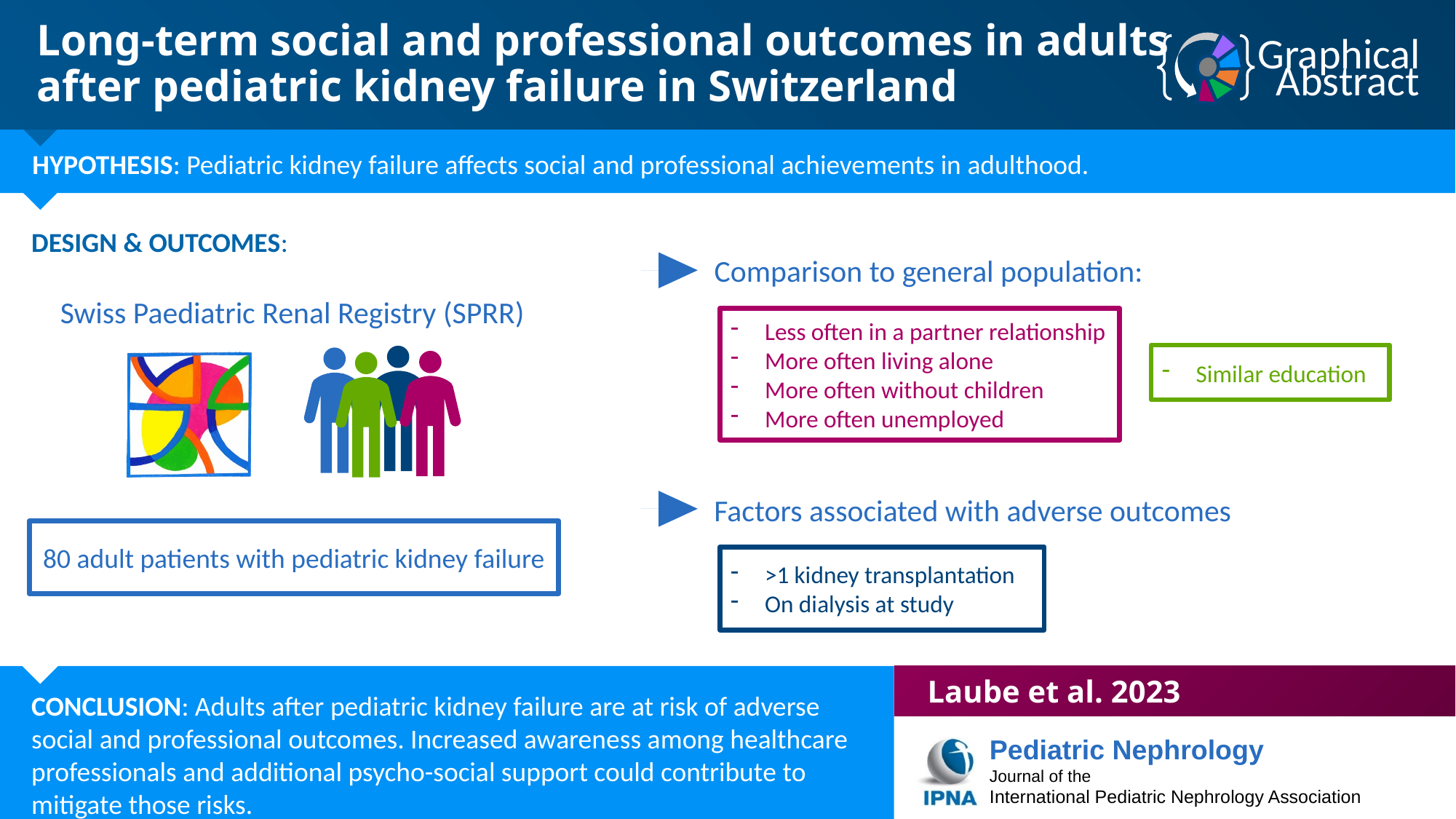

Long-term social and professional outcomes in adults
after pediatric kidney failure in Switzerland
HYPOTHESIS: Pediatric kidney failure affects social and professional achievements in adulthood.
DESIGN & OUTCOMES:
Comparison to general population:
Swiss Paediatric Renal Registry (SPRR)
Less often in a partner relationship
More often living alone
More often without children
More often unemployed
Similar education
Factors associated with adverse outcomes
80 adult patients with pediatric kidney failure
>1 kidney transplantation
On dialysis at study
Laube et al. 2023
CONCLUSION: Adults after pediatric kidney failure are at risk of adverse social and professional outcomes. Increased awareness among healthcare professionals and additional psycho-social support could contribute to mitigate those risks.
