# Supplementary material for: Long-term social and professional outcomes in adults after pediatric kidney failure
Source: Pediatr Nephrol. 2023 Jun 17;38(11):3769–77. doi: 10.1007/s00467-023-06029-2 (PMC10514133; doi:10.1007/s00467-023-06029-2)
Supplement: Supplementary file 2 — Supplementary file 1 (DOCX 66 KB) [file 467_2023_6029_MOESM2_ESM.docx]

**Pediatric Nephrology**

**Long-term social and professional outcomes in adults after pediatric kidney failure in Switzerland**

**Authors and Affiliations**

Guido F Laube^1,2^, Marc-Andrea Heinzelmann^2,3^, Katharina Roser^4^, Claudia E Kuehni^2,3,5^, Luzius Mader^2,3^, on behalf of the Swiss Pediatric Renal Registry (SPRR) group

^1^Department of Pediatrics, Hospital Baden, Baden, Switzerland;

^2^Swiss Pediatric Renal Registry, Child and Adolescent Health Research Group, Institute of Social and Preventive Medicine, University of Bern, Bern, Switzerland;

^3^Child and Adolescent Health Research Group, Institute of Social and Preventive Medicine, University of Bern, Bern, Switzerland;

^4^Department of Health Sciences and Medicine, University of Lucerne, Lucerne, Switzerland;

^5^Department of Pediatrics, University Children's Hospital Bern, Bern, Switzerland.

**Corresponding author**

Luzius Mader, Child and Adolescent Health Research Group, Institute of Social and Preventive Medicine (ISPM), University of Bern, Mittelstrasse 43, 3012 Bern, Switzerland.

Phone: +41 31 684 33 47, email: [luzius.mader@ispm.unibe.ch](mailto:luzius.mader@ispm.unibe.ch)

**Online Resource 1.** Comparison of participants and non-participants, based on information from the SPRR

|  | | Participants  (n=80) | Non-participants  (n=86) | p-value^a^ |
| --- | --- | --- | --- | --- |
| *Mean age at study in years (SD)* | | 39 (10) | 40 (11) | 0.296 |
| *Sex* | |  |  | 0.925 |
|  | Male | 45 (56) | 49 (57) |  |
|  | Female | 35 (44) | 37 (43) |  |
| *Type of kidney disease* | |  |  | 0.900 |
|  | Congenital anomalies kidney/ urinary tract | 29 (36) | 34 (40) |  |
|  | Monogenetic hereditary diseases | 35 (44) | 35 (41) |  |
|  | Acquired diseases | 16 (20) | 17 (20) |  |
| *Mean age at first KRT in years (SD)* | | 10 (5) | 11 (4) | 0.164 |
| *Mean duration of KRT in years (SD)* | | 28 (9) | 29 (10) | 0.655 |

Abbreviations: KRT, kidney replacement therapy; SD, standard deviation; SPRR, Swiss Paediatric Renal Registry.

^a^P-value from chi-squared tests (categorical variables) or two-sided t tests (continuous variables) comparing participants and non-participants.

**Online resource 2.** Socio-demographic and clinical determinants of adverse social and professional outcomes from univariable logistic regression models in SPRR participants

|  | | SPRR study population (n=80) | | | | | |
| --- | --- | --- | --- | --- | --- | --- | --- |
|  | | Partner relationship  (n=79) | Living situation  (n=80) | Having children  (n=80) | Educational achievement  (n=76) | Employment status  (n=80) |  |
| Explanatory variables | | OR (95%-CI)^a^ | OR (95%-CI)^a^ | OR (95%-CI)^a^ | OR (95%-CI)^a^ | OR (95%-CI)^a^ |  |
| *Age at study [years]* | | 0.98 (0.94-1.02) | 1.02 (0.97-1.07) | 0.95 (0.89-1.01) | 0.98 (0.94-1.02) | 1.03 (0.98-1.08) |  |
| *Sex* | |  |  |  |  |  |  |
|  | Male | ref | ref | ref | ref | ref |  |
|  | Female | 0.8 (0.3-2.0) | 0.6 (0.2-1.5) | 0.4 (0.1-1.5) | 0.8 (0.3-2.1) | 1.1 (0.4-3.0) |  |
| *Attendance of special school* | |  |  |  |  |  |  |
|  | No | ref | ref | ref | ref | ref |  |
|  | Yes | **3.8 (1.2-11.8)** | 0.6 (0.2-1.9) | 1.7 (0.3-8.8) | **22.7 (2.8-182.7)** | 1.7 (0.6-5.2) |  |
| *Educational support during hospital stays* | |  |  |  |  |  |  |
|  | No | ref | ref | ref | ref | ref |  |
|  | Yes | 0.6 (0.2-1.4) | 0.9 (0.4-2.4) | 0.3 (0.1-1.4) | 1.5 (0.6-3.7) | 0.5 (0.2-1.5) |  |
| *Type of kidney disease* | |  |  |  |  |  |  |
|  | CAKUT | ref | ref | ref | ref | ref |  |
|  | Monogenetic hereditary diseases | 1.0 (0.4-2.6) | 0.4 (0.1-1.2) | 1.0 (0.3-3.7) | 0.6 (0.2-1.7) | 0.5 (0.2-1.8) |  |
|  | Acquired diseases | 0.9 (0.3-3.2) | 0.4 (0.1-1.6) | n.e. | 0.7 (0.2-2.6) | 1.6 (0.4-5.8) |  |
| *Age at first KRT* | |  |  |  |  |  |  |
|  | <10 years | ref | ref | ref | ref | ref |  |
|  | ≥10 years | 0.8 (0.3-2.0) | 0.7 (0.3-1.8) | 0.3 (0.1-1.4) | 0.5 (0.2-1.4) | 1.3 (0.5-3.8) |  |
| *Duration of KRT* | |  |  |  |  |  |  |
|  | <25 years | ref | ref | ref | ref | ref |  |
|  | ≥25 years | 0.7 (0.3-1.8) | 1.8 (0.7-4.8) | 1.3 (0.4-4.7) | 1.0 (0.4-2.6) | 1.0 (0.4-2.8) |  |
| *Type of KRT at study* | |  |  |  |  |  |  |
|  | Transplantation | ref | ref | ref | ref | ref |  |
|  | Dialysis | 0.8 (0.2-3.0) | 0.9 (0.2-3.7) | 1.4 (0.2-12.4) | 2.1 (0.5-9.0) | **5.7 (1.4-22.9)** |  |
| *Number of transplants* | |  |  |  |  |  |  |
|  | 1 transplant | ref | ref | ref | ref | ref |  |
|  | >1 transplant | 0.7 (0.3-1.6) | 1.2 (0.5-3.0) | 0.2 (0.1-1.0) | 1.8 (0.7-4.5) | 1.4 (0.5-3.9) |  |
| *Height [in cm]* | | 0.99 (0.95-1.04) | 1.04 (0.98-1.10) | 1.04 (0.97-1.09) | 0.98 (0.93-1.02) | 0.95 (0.90-1.00) |  |

Abbreviations: CAKUT, congenital anomalies of the kidney/ urinary tract CI, confidence interval; KRT, kidney replacement therapy; n.e., not estimated due to empty cells; OR, odds ratio; ref, reference group; SPRR, Swiss Paediatric Renal Registry. Statistically significant associations at p<0.05 are highlighted in bold.

^a^Odds ratio from logistic regression models: OR>1 indicate a higher likelihood of not having a partner relationship, living alone, not having children, having a lower education, or being unemployed. OR<1 indicate a lower likelihood of not having a partner relationship, living alone, not having children, having a lower education, or being unemployed.
